# Occupational Depression, Cognitive Performance, and Task Appreciation: A Study Based on Raven’s Advanced Progressive Matrices

**DOI:** 10.3389/fpsyg.2021.695539

**Published:** 2021-09-20

**Authors:** Renzo Bianchi, Irvin Sam Schonfeld

**Affiliations:** ^1^Institute of Work and Organizational Psychology, University of Neuchâtel, Neuchâtel, Switzerland; ^2^Department of Psychology, The City College of the City University of New York, New York, NY, United States

**Keywords:** Occupational Depression Inventory, cognition, job performance, mood, work stress, positive feedback loop, helplessness, burnout

## Abstract

The Occupational Depression Inventory (ODI) was recently developed to assess depressive symptoms that individuals specifically attribute to their work. Research on the criterion validity of the instrument is still in its infancy. In this study, we examined whether the ODI predicted performance on, and appreciation of, a cognitively challenging test. In light of the link established between clinical depression and neuropsychological impairment, and considering that individuals with depressive symptoms are more likely to feel helpless under challenging circumstances, we hypothesized that occupational depression would be associated with poorer cognitive performance and a darkened appreciation of the task undertaken. We relied on a sample of 1,359 educational staff members. We used an abridged version of Raven’s Advanced Progressive Matrices (RAPMs) as a cognitively challenging task and measure of cognitive performance. RAPMs assess so-called eductive ability (meaning-making and problem-solving abilities) through items of various degrees of difficulty. Transient mood was assessed with a three-item measure before RAPMs were administered. Task appreciation was assessed with a single-item measure after the completion of RAPMs. We found occupational depression to be negatively linked to cognitive performance. A two-step cluster analysis, in which ODI and RAPMs scores were used as classifiers, revealed two profiles of respondents. The first profile was characterized by relatively low levels of depressive symptoms and high levels of cognitive performance; the second profile, by relatively high levels of depressive symptoms and low levels of cognitive performance. The two profiles differed strongly from one another, as indexed by Cohen’s *d*s of 2.492 regarding depressive symptoms and 1.263 regarding cognitive performance. As anticipated, occupational depression predicted a darkened appreciation of the test. The association remained statistically significant, and largely unchanged, controlling for pretest mood and test performance. The highest levels of depressive symptoms were observed among individuals evaluating the task as “frustrating” and “discouraging.” Our study suggests that occupational depression predicts poorer cognitive performance and a negativized experience of cognitive challenge. Such features may be part of a self-sustaining loop fostering the maintenance of depressive symptoms. The extent to which the ODI predicts performance in the work context needs to be investigated.

## Introduction

The Occupational Depression Inventory (ODI) was recently developed to assess depressive symptoms that individuals specifically attribute to their work (Bianchi and Schonfeld, 2020, 2021a,b). The ODI references the nine diagnostic symptom criteria for major depressive disorder found in the *Diagnostic and statistical manual of mental disorders* (5th ed.; *DSM-5*; American Psychiatric Association, 2013). The ODI thus assesses anhedonia, depressed mood, sleep alterations, fatigue/loss of energy, appetite alterations, feelings of worthlessness, cognitive impairment, psychomotor alterations, and suicidal ideation. The ODI was devised to overcome limitations of current measures of job-related distress, most notably, burnout measures (Bianchi et al., 2017, 2020, 2021; Rotenstein et al., 2018; Schwenk and Gold, 2018; Schonfeld et al., 2019a,b; Verkuilen et al., 2020; Vinkers and Schaafsma, 2021). Available evidence indicates that the ODI has excellent psychometric and structural properties and constitutes a promising tool for occupational health specialists (Bianchi and Schonfeld, 2020, 2021a,b). The ODI can be found in Bianchi and Schonfeld’s (2020) original article on the instrument and on the website of the Society for Occupational Health Psychology.^1^

Among individuals with no noticeable vulnerability to depression, the development of depressive symptoms and disorders has been critically linked to situations of unresolvable stress (Willner et al., 2013; Moscarello and Hartley, 2017; Grahek et al., 2019). Situations of unresolvable stress are situations in which individuals cannot take effective action to neutralize the stressors encountered and are, therefore, reduced to passively enduring the adverse effects of those stressors. Put differently, individuals are trapped in the face of stressors perceived as uncontrollable (Laborit, 1974, 1986; Seligman, 1975; Breier et al., 1987; Abramson et al., 1989; Dohrenwend, 2000; Pryce et al., 2011; Bhanji et al., 2016). An important characteristic of such states of helplessness and resignation is that they generalize to new challenging situations (Seligman, 1975; Pryce et al., 2011; Moscarello and Hartley, 2017). It is noteworthy that dealing successfully with stress-eliciting situations is fundamentally rewarding—to act effectively is to act rewardingly (Pryce et al., 2011; Rolls, 2016; Bondy et al., 2021). Unresolvable stress thus involves reward-hindering and punitive states. Such states are reflected in a disruption of allostasis and a promotion of allostatic load (McEwen, 2000; Honkalampi et al., 2021). The imbalance between positive affect and negative affect lies at the heart of the etiology of depression. Over the last decades, interest in depression in the context of work has grown considerably (Schonfeld and Chang, 2017). Many workers find themselves trapped in situations where (a) they perceive the job stressors that assail them as unmanageable and unchangeable and (b) they consider leaving their job impossible because such an option would result, for instance, in pension loss, long-term unemployment, and/or financial ruin—i.e., in situations evaluated as even more undesirable than their current situation. The ODI is intended to help occupational health specialists deal with job-ascribed forms of depression more effectively.

A key aspect of depressive symptomatology lies in altered cognitive functioning, in terms of (a) processing efficacy and (b) information selection and construction. Regarding information selection and construction, there is ample evidence that depressive symptoms are associated with so-called biases in favor of “negative” information (or to the detriment of “positive” information) at the levels of attention, interpretation, and memory (Everaert et al., 2014; Bianchi and Laurent, 2015; Bianchi and Schonfeld, 2016; Bianchi et al., 2018; Bianchi and da Silva Nogueira, 2019; LeMoult and Gotlib, 2019). Regarding processing efficacy, depression has been associated with pervasive impairment, for instance, at the level of executive functions (Snyder, 2013; Rock et al., 2014; Ahern and Semkovska, 2017; Semkovska et al., 2019; Pettersson et al., 2021). These findings are consistent with the difficulties in concentration and decision-making frequently reported by depressed patients in clinical settings as well as with the fact that cognitive impairment constitutes a diagnostic criterion for major depressive disorder (American Psychiatric Association, 2013). Cognitive alterations have been regarded as a central mediator of the link between clinical depression and psychosocial impairment, including deteriorations in job performance (McIntyre et al., 2013; Lam et al., 2014; Pan et al., 2019).

Although the ODI has displayed excellent psychometric and structural properties to date (Bianchi and Schonfeld, 2020), research on the criterion validity of the instrument is still in its infancy. In the present study, we inquired into the criterion validity of the ODI by examining whether the ODI predicted performance on, and appreciation of, a cognitively challenging test. Investigating the criterion validity of the ODI in relation to cognitive functioning appears to be particularly relevant given that, as mentioned above, cognitive alterations are assumed to play a mediating role in the relationship between clinical depression and psychosocial impairment, notably from the standpoint of worker performance (McIntyre et al., 2013; Lam et al., 2014; Pan et al., 2019). In light of the well-established link between clinical depression and neuropsychological impairment (Snyder, 2013; Rock et al., 2014; Ahern and Semkovska, 2017; Semkovska et al., 2019; Pettersson et al., 2021), and considering that individuals with depressive symptoms are more likely to feel helpless under challenging circumstances (Seligman, 1975; Pryce et al., 2011), we hypothesized that occupational depression would be associated with poorer cognitive performance and a darkened appreciation of the task undertaken. Learning more about the criterion validity of the ODI is important in order to further estimate the (practical) utility of the instrument for occupational health specialists.

## Materials and Methods

### Study Sample

This study involved a convenience sample of educational staff members (e.g., teachers, principals, librarians) employed in K-12 schools in France. Educational staff members tend to be exposed to a variety of chronic work stressors (e.g., verbal intimidation and physical violence; Schonfeld, 2006; Reddy et al., 2013; Longobardi et al., 2019) fostering the development of depressive symptoms (e.g., Schonfeld, 2001; Galand et al., 2007). Assuming that variability in the extent to which educators are subject to chronic work stressors is related to variability in ODI scores, we expected to observe a range of scores from ‘‘low’’ to ‘‘medium’’ to ‘‘high’’ on the ODI, thus providing the variance needed for our analyses. Participants were reached through email contacts with school administrators and directors. Participation in the study was voluntary and without compensation. Consent to participate was requested. Our study complied with the ethical standards of the institutional review board of the University of Neuchâtel. The study was conducted online using Qualtrics.^2^

Our survey included the ODI, an abridged version of Raven’s Advanced Progressive Matrices (RAPMs), measures of transient mood and task appreciation, and sociodemographic items. At the very end of the survey, participants were asked if they had completed the RAPMs alone and without help. A negative response to this item was eliminatory. Of the 1,743 participants who had completed the survey, 323 were excluded on this basis (18.531% of the initial sample). Of the 1,420 remaining participants, 61 were eliminated (4.296% of the corrected sample) due to survey completion durations exceeding 60 min. Durations exceeding 60 min were considered aberrantly long because (a) the completion of RAPMs—presumably the most time-consuming part of the study—was not expected to take more than 20 min given the use of an abbreviated version (Arthur and Day, 1994; Raven et al., 1998) and (b) the rest of the survey comprised a limited number of relatively brief measures (e.g., the ODI). A 60-min cutoff thus allowed us to exclude participants with completion durations reflecting extreme degrees of distraction (e.g., durations signaling survey completion on more than a day) without being overly restrictive. The most rapid respondent completed the survey in 6.133 min. The respondent in question displayed an above-average score of 0.833 on the RAPMs, suggesting that a lower boundary for aberrant response duration was not needed. A total of 1,359 participants eventually constituted the study sample (mean completion time = 15.332, *SD* = 7.585). Participants’ mean age was 45.309 (*SD* = 9.876). About 85% of the participants were women (*n* = 1,149), consistent with the large overrepresentation of women in the French K-12 academic system.^3^

### Measures of Interest

The pivotal study measure was the ODI (Bianchi and Schonfeld, 2020, 2021a,b). The ODI comprises nine core symptom items rated on a frequency scale from 0 (“never or almost never”) to 3 (“nearly every day”). All of these items are negatively worded. As previously mentioned, the ODI was designed with reference to the nine diagnostic symptom criteria for major depressive disorder found in the *DSM-5* (American Psychiatric Association, 2013). The ODI includes six anhedonic-somatic symptom items (anhedonia, sleep alterations, fatigue/loss of energy, appetite alterations, cognitive impairment, and psychomotor alterations) and three dysphoric symptom items (depressed mood, feelings of worthlessness, and suicidal ideation). A sample item is: “My experience at work made me feel like a failure.” In this study, the ODI had a McDonald’s ω of 0.933. As per Bianchi and Schonfeld (2020), we reexamined the factorial structure of the ODI based on exploratory structural equation modeling (ESEM) bifactor analysis. We did so in Mplus 8 (Muthén and Muthén, 1998–2017). We treated the items as ordinal. We used the WLSMV estimator and relied on a partially specified target (orthogonal) rotation (Marsh et al., 2014; Li, 2016). Two bifactors were considered in addition to the general “Occupational Depression” factor. One was an “Anhedonic-Somatic” bifactor, reflecting the six anhedonic-somatic symptom items of the ODI. The other was a “Dysphoric” bifactor, reflecting the three dysphoric symptom items of the ODI. The model showed a satisfactory fit: RMSEA = 0.000 (90% confidence interval: 0.000, 0.026); CFI = 1.000; TLI = 1.000, WRMR = 0.271; χ^2^(12) = 11.185. The general factor accounted for 88.691% of the common variance extracted, a proportion indicative of essential unidimensionality (Rodriguez et al., 2016). Consistent with these findings, a confirmatory factor analysis (CFA) in which we treated the items as ordinal and used the WLSMV estimator showed that a one-factor model had an acceptable fit: RMSEA = 0.071 (90% confidence interval: 0.062, 0.080); CFI = 0.988; TLI = 0.985; SRMR = 0.041; χ^2^(27) = 211.159. The ODI includes a subsidiary question related to turnover intention: “If you have encountered at least some of the problems mentioned above, do these problems lead you to consider leaving your current job or position?” Three response options are provided: “yes,” “no,” and “I don’t know.” In this study, nearly one third of the participants (*n* = 440) answered this question in the affirmative.

**FIGURE 1:**
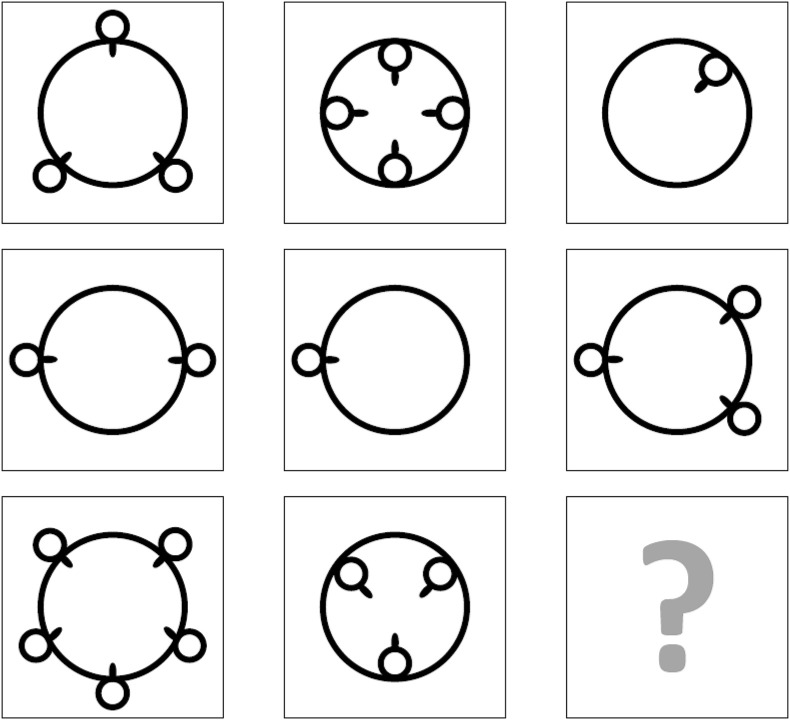
Sample matrix adapted from Raven’s Advanced Progressive Matrices.

We used a 12-item version of RAPMs as a cognitively challenging task and a measure of cognitive performance (Arthur and Day, 1994; Raven et al., 1998). RAPMs were designed to assess so-called eductive ability (also known as meaning-making ability), i.e., the ability to generate order and infer valid structuring rules (Raven and Raven, 2003). The stronger an individual’s eductive ability, the higher the probability for this individual to decipher complex stimuli. RAPMs are non-verbal in nature. Each matrix is made up of three rows and three columns. In each of the matrices, eight shapes are present and one shape is missing. The participant’s goal is to identify the missing shape that logically completes the set. A sample matrix adapted from RAPMs is displayed in Figure 1. RAPMs showed a McDonald’s ω of 0.916 in this study. Relying on Mplus 8 (Muthén and Muthén, 1998–2017), we submitted RAPMs to a CFA in which the items were treated as categorical and the WLSMV estimator was used. A one-factor model showed a satisfactory fit: RMSEA = 0.024 (90% confidence interval: 0.016, 0.032); CFI = 0.989; TLI = 0.987; SRMR = 0.048; χ^2^(54) = 97.771. The RAPMs session was divided into two blocks. The first block, consisting of eight relatively easy matrices, was a training block intended to familiarize participants with the test and its interface. The second block, consisting of four relatively difficult matrices, was our target block for examining individuals’ cognitive performance. For each matrix, participants were presented with eight response options and asked to select the answer they considered correct. A correct answer was coded 1 and any incorrect answer was coded 0, leading to a mean score between 0 and 1. Detailed descriptive statistics for RAPMs are available in Table 1.

**TABLE 1:** Descriptive statistics for Raven’s Advanced Progressive Matrices (RAPMs).

	M1	M2	M3	M4	M5	M6	M7	M8	Training	M9	M10	M11	M12	Test	RAPMs
*M*	0.939	0.924	0.923	0.892	0.884	0.875	0.828	0.723	0.873	0.620	0.607	0.489	0.314	0.507	0.751
Median	1	1	1	1	1	1	1	1	1.000	1	1	0	0	0.500	0.833
Mode	1	1	1	1	1	1	1	1	1.000	1	1	0	0	0.500	0.833
*SD*	0.240	0.265	0.267	0.311	0.321	0.331	0.378	0.448	0.205	0.486	0.489	0.500	0.464	0.294	0.200
Skewness (*SE* = 0.066)	–3.670	–3.209	–3.170	–2.526	–2.397	–2.269	–1.738	–0.995	–2.470	–0.493	–0.439	0.046	0.801	–0.065	–1.516
Kurtosis (SE = 0.133)	11.485	8.311	8.061	4.387	3.751	3.153	1.024	–1.011	6.632	–1.759	–1.810	–2.001	–1.360	–0.818	2.824
Minimum	0	0	0	0	0	0	0	0	0.000	0	0	0	0	0.000	0.000
Maximum	1	1	1	1	1	1	1	1	1.000	1	1	1	1	1.000	1.000
% correct response	93.893	92.421	92.274	89.183	88.374	87.491	82.781	72.259	87.334	61.957	60.706	48.859	31.420	50.736	75.135

*N = 1,359 (no missing values). Mean scores fall between 0 and 1. M1–M12: matrices 1–12; Training, RAPMs training section; Test, RAPMs test section; M, mean; SD, standard deviation; SE, standard error.*

**FIGURE 2:**
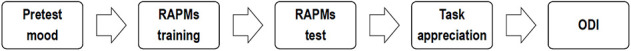
Summary of the study procedure (RAPMs: Raven’s Advanced Progressive Matrices; ODI: Occupational Depression Inventory).

We assessed transient mood with a three-item measure focusing on positive mood, dejected mood, and irritable mood (van Eck et al., 1998). We relied on the following items: “I feel fine, I am in a good mood”; “I feel down, demoralized”; “I feel angry, irritated.” Items were rated on a scale from 1 (“strongly agree”) to 5 (“strongly disagree”). The first item (“I feel fine, I am in a good mood”) was reverse-coded when computing mean scores. Higher mean scores were, therefore, indicative of more negative mood states. Transient mood was evaluated before RAPMs were administered (see Figure 2 for a summary of the study protocol). McDonald’s ω was 0.892.^4^

**FIGURE 3:**
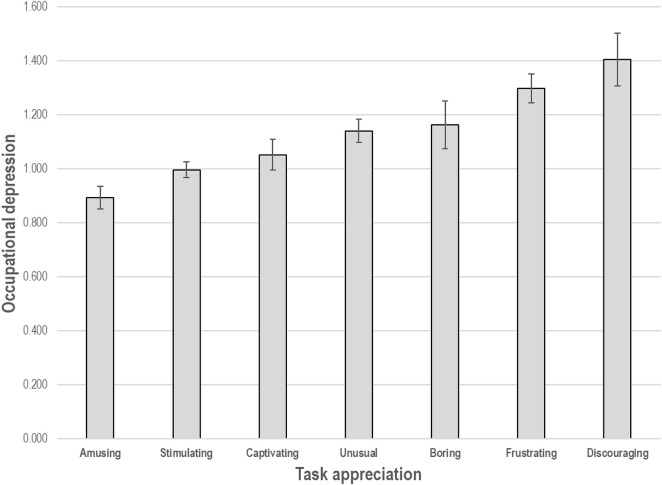
Mean score on the Occupational Depression Inventory (± 1 standard error) per task appreciation category.

In order to assess task appreciation, we asked our participants to indicate which, of seven terms, best described the test they had completed. The terms were divided into: three positive expressions---amusing, stimulating, and captivating; three negative expressions---boring, frustrating, and discouraging; and one expression deemed to be neutral---unusual.^5^ ODI scores associated with each appreciation term are displayed in Figure 3. We recoded the terms into three categories—0 for negative expressions, 1 for the neutral expression, and 2 for positive expressions for our analyses. Higher task appreciation scores were thus reflective of more favorable evaluations of the test. Task appreciation was assessed right after RAPMs were completed.

### Data Analyses

We first examined the link between occupational depression and cognitive performance using correlational analysis, hierarchical linear regression analysis, and two-step cluster analysis. Two-step cluster analysis allows the investigator to identify individual profiles based on characteristics of interest, called classifiers, and is thus anchored in a person-centered approach (as opposed to a variable-centered approach; Howard and Hoffman, 2017; for specific examples, see Ahola et al., 2014; Bianchi et al., 2015; Vicent-Gil et al., 2020). We employed occupational depression and cognitive performance as classifiers. We relied on a Euclidean distance measure and used Schwarz’s Bayesian clustering criterion. We did not prespecify the number of clusters. Model fit was estimated with the Silhouette coefficient, an index of cluster cohesion and separation. We additionally used Welch’s analysis of variance (ANOVA), analysis of covariance, and Pearson’s chi-squared test to examine the characteristics of our clusters.

We then focused on the link between occupational depression and task appreciation relying on correlational analysis and hierarchical linear regression analysis. We anticipated that pretest mood and cognitive performance would need to be controlled given likely links between these two variables and both our main predictor (occupational depression) and our outcome of interest (task appreciation).

## Results

### Occupational Depression and Cognitive Performance

Occupational depression correlated negatively with cognitive performance, ρ = -0.093, *p* = 0.001, and *r* = -0.098, *p* < 0.001 (Table 2). The association remained statistically significant controlling for age, sex, and pretest transient mood in hierarchical linear regression analysis (Supplementary Table 1). When corrected for measurement error within a confirmatory factor analytic framework, the correlation between the Occupational Depression and Eductive Ability factors reached -0.155, *p* < 0.001 [RMSEA = 0.034 (90% confidence interval: 0.028, 0.041), CFI = 0.994, TLI = 0.993, SRMR = 0.034]. The raw correlation between occupational depression and pretest mood was large, ρ = 0.557, *p* < 0.001, and *r* = 0.568, *p* < 0.001.

**TABLE 2:** Spearman and Pearson correlations among the main study variables.

		*M*	*SD*	Min.	Max.	1.	2.	3.	4.	5.	6.
1.	Occupational depression	1.067	0.675	0.000	3.000	—	–0.093	–0.057	–0.080	0.557	–0.180
2.	Cognitive performance	0.507	0.294	0.000	1.000	–0.098	—	–0.055	0.107	–0.086	0.301
3.	Age	45.309	9.876	22	67	–0.053	–0.041	—	0.071	0.054	–0.014
4.	Sex	0.155	0.362	0	1	–0.080	0.105	0.069	—	0.011	0.029
5.	Pretest mood	2.639	1.036	1.000	5.000	0.568	–0.086	0.054	0.011	—	–0.110
6.	Task appreciation	1.427	0.801	0.000	2.000	–0.179	0.301	0.015	0.029	–0.110	—

*N = 1,359. Spearman correlations are displayed above the diagonal and Pearson correlations, below the diagonal. Any correlation coefficient the absolute value of which ≥ 0.053 is statistically significant at p < 0.05. Regarding pretest mood, higher scores are reflective of a more negative mood. Sex was coded 0 for women and 1 for men. M, mean; SD, standard deviation.*

Two-step cluster analysis revealed two individual profiles. The first cluster (Cluster 1; *n* = 1,214) involved individuals with relatively low ODI scores (*M* = 0.935, *SD* = 0.572) and relatively high cognitive performance (*M* = 0.541, *SD* = 0.284). The second cluster (Cluster 2; *n* = 145) involved individuals with relatively high ODI scores (*M* = 2.172, *SD* = 0.407) and relatively low cognitive performance (*M* = 0.226, *SD* = 0.209). The model showed a satisfactory fit, with a Silhouette coefficient of 0.4 (Vicent-Gil et al., 2020). Welch’s ANOVAs revealed that the two clusters differed substantially from one another in terms of both ODI scores, *^*a*^F*(1, 218.360) = 1083.277, *p* < 0.001, Cohen’s *d* = 2.492, and cognitive performance, *^*a*^F*(1, 213.427) = 269.956, *p* < 0.001, Cohen’s *d* = 1.263. Characteristics of the two clusters (in terms of age, sex, pretest mood, and task appreciation) are presented in Table 3.

**TABLE 3:** Between-cluster comparisons.

	Cluster 1 (*n* = 1,214)		Cluster 2 (*n* = 145)		Welch’s ANOVA			
			
	*M*	*SD*	*M*	*SD*	*F*	df	*p*	Cohen’s *d*
Age	45.238	9.815	45.903	10.396	0.537	1, 176.055	0.465	0.066
Pretest mood	2.530	0.989	3.552	0.969	143.240	1, 181.760	(<0.001	1.044
Task appreciation	1.483	0.772	0.959	0.881	47.006	1, 171.466	<0.001	0.633

	**Cluster 1 (*n* = 1,214)**		**Cluster 2 (*n* = 145)**		**Pearson’s chi-squared test**			
			
	**% female**		**% female**		***X* ^2^**	**df**	***p***	**Phi**

Sex	83.526		93.103		9.095	1	0.003	0.082

*Cluster construction was based on two continuous classifiers, occupational depression and cognitive performance. Between-cluster differences pertaining to task appreciation remained statistically significant controlling for pretest mood (as well as age and sex) in analyses of covariance. We did not include a Bonferroni correction given the controversies surrounding its use (e.g., Perneger, 1998). ANOVA, analysis of variance; df, degrees of freedom; M, mean; SD, standard deviation.*

### Occupational Depression and Task Appreciation

Correlational analyses indicated that occupational depression was associated with a darkened appreciation of the task undertaken, ρ = -0.180, *p* < 0.001, and *r* = -0.179, *p* < 0.001 (Table 2). Hierarchical linear regression analysis showed that the association remained statistically significant, and largely unchanged, controlling for pretest mood and cognitive performance (Table 4).^6^ Pretest mood did not interact with occupational depression in predicting task appreciation, whether controlling for cognitive performance (*p* = 0.685) or not (*p* = 0.493).

**TABLE 4:** Summary of hierarchical linear regression analysis—occupational depression predicting task appreciation.

	Task appreciation			
	
	β	*t*	*p*	Adj. *R*^2^
Step 1				0.011
Pretest mood	–0.110	–4.087	0.000	
Step 2				0.097
Pretest mood	–0.085	–3.282	0.001	
Cognitive performance	0.294	11.357	0.000	
Step 3				0.111
Pretest mood	0.000	–0.007	0.994	
Cognitive performance	0.287	11.137	0.000	
Occupational depression	–0.150	–4.824	0.000	

*N = 1,359. Regarding pretest mood, higher scores are reflective of a more negative mood. No variance inflation factor exceeded 1.482, suggesting that multicollinearity was not an issue.*

## Discussion

The present study examined whether the ODI predicted performance on, and appreciation of, a cognitively challenging test. In so doing, our goal was to learn more about the criterion validity of the ODI. In light of the well-established link between clinical depression and neuropsychological impairment (Snyder, 2013; Rock et al., 2014; Ahern and Semkovska, 2017; Semkovska et al., 2019; Pettersson et al., 2021), and considering that individuals with depressive symptoms are more likely to feel helpless under challenging circumstances (Seligman, 1975; Pryce et al., 2011), we hypothesized that occupational depression would be associated with (a) poorer cognitive performance and (b) a darkened appreciation of the task undertaken.

Consistent with our first hypothesis, we found occupational depression to be negatively associated with cognitive performance. The correlations that we obtained between occupational depression and cognitive performance fall in the range of the documented correlations between RAPMs and self-reported measures of depressive symptoms such as the Center for Epidemiological Studies-Depression scale (du Pont et al., 2020). By adopting a person-centered, cluster-analytic approach to the link between occupational depression and cognitive performance, we were able to identify two distinct individual profiles. The first profile was characterized by relatively low levels of work-attributed depressive symptoms and high levels of cognitive performance whereas the second profile was marked by relatively high levels of work-attributed depressive symptoms and low levels of cognitive performance. Between-profile differences were large in terms of both occupational depression and cognitive performance, as indexed by Cohen’s *d*s ≥ 1.263. Such effect sizes are consistent with those found in cluster-analytic research pertaining to neuropsychological functioning in clinical depression (Vicent-Gil et al., 2020). Our findings underline the utility of combining variable- and person-centered approaches in depression research. Our results dovetail with those pertaining to the link between clinical depression and neuropsychological impairment (Snyder, 2013; Rock et al., 2014; Ahern and Semkovska, 2017; Semkovska et al., 2019; Pettersson et al., 2021).

Consistent with our second hypothesis, we found occupational depression to be associated with a darkened appreciation of the test. Importantly, the association remained statistically significant controlling not only for pretest mood but also for test performance, suggesting that occupational depression darkens individuals’ view of the task undertaken in a way that is partly independent of their ability to handle the task in question. These results are in keeping with the well-established finding that depression involves a negative filter in the processing of information (Bianchi et al., 2018; Bianchi and da Silva Nogueira, 2019; LeMoult and Gotlib, 2019). In addition, we found that individuals evaluating the task as “frustrating” and “discouraging” were also those who exhibited the highest levels of depressive symptoms. This finding resonates with the observation that feelings of helplessness and resignation constitute a hallmark of depression (Seligman, 1975; Pryce et al., 2011).

In keeping with Bianchi and Schonfeld’s (2020) findings regarding the psychometric and structural properties of the ODI, we found the ODI to exhibit strong reliability, high factorial validity, and essential unidimensionality. In Bianchi and Schonfeld’s (2020) study, the ODI manifested moderate to large correlations, in the expected direction, with a variety of self-reported measures, including self-reported measures of job satisfaction, dedication to work, willingness to stay in the job, social support in work life, active search for another job/position, trait anxiety, general health status, and life satisfaction. By documenting a link between individuals’ scores on the ODI and RAPMs, this study extends our knowledge of the ODI’s properties by suggesting that the ODI is predictive of “objective,” test-assessed cognitive performance. This constitutes an important step in the examination of the predictive value and practical utility of the ODI.

At least five limitations to the present study can be underlined. First, although relatively large in size (*N* = 1,359), our study sample was a convenience sample. Because our participants were self-selected, the representativeness of our study sample vis-à-vis its population of reference is not clear (e.g., in terms of basic demographics). Second, our study sample consisted only of educational staff members. The extent to which our findings can be generalized to other occupational groups remains to be elucidated. Education professionals may, for instance, better compensate for the undermining effects of depressive symptoms on cognitive performance than other occupational groups thanks to above-average cognitive reserves (Stern, 2009). Third, we assessed participants’ appreciation of the task undertaken using a single-item measure. Although single-item measures can do a fine job under a number of circumstances (e.g., Friedman et al., 2017) and are much more reliable than often assumed (Bowling, 2005; Mõttus and Rozgonjuk, 2021), using multiple-item measures is generally preferable. Fourth, even if RAPMs mobilize a wide array of cognitive abilities, the inclusion of a broader set of cognitive tests would have been a plus. Fifth, our study was conducted online, which involves a lesser degree of control over test-taking conditions compared to classical laboratory settings. Reassuringly, however, web-based studies have proved valid and reliable in psychological research and beyond (Horton et al., 2011; Gosling and Mason, 2015; Anwyl-Irvine et al., 2021). We also note that, as mentioned earlier, we took measures to identify and exclude careless participants.

## Conclusion

The present study suggests that the ODI predicts poorer cognitive performance and a negativized experience of cognitively challenging circumstances. Such features may be part of a self-sustaining loop fostering the maintenance of depressive symptoms. Depressive symptoms may render cognitive challenges both more difficult and more unpleasant to handle, which, in turn, may feed depressive symptoms further. Both replication studies and studies assessing performance directly in the work context are needed to learn more about the criterion validity of the ODI.

## Data Availability Statement

The datasets presented in this article are not readily available because of confidentiality and data protection commitments. Requests to access the datasets should be directed to corresponding author.

## Ethics Statement

Ethical review and approval was not required for the study on human participants in accordance with the local legislation and institutional requirements. The patients/participants provided their written informed consent to participate in this study.

## Author Contributions

RB developed the study concept, designed the study protocol, collected the data, and drafted the manuscript. RB and IS performed data analysis. Both authors took part in result interpretation, reviewed and edited several versions of the manuscript. Both authors provided critical revisions and approved the final version of the manuscript for submission.

## Conflict of Interest

The authors declare that the research was conducted in the absence of any commercial or financial relationships that could be construed as a potential conflict of interest.

## Publisher’s Note

All claims expressed in this article are solely those of the authors and do not necessarily represent those of their affiliated organizations, or those of the publisher, the editors and the reviewers. Any product that may be evaluated in this article, or claim that may be made by its manufacturer, is not guaranteed or endorsed by the publisher.

## Footnotes

1
https://sohp-online.org/resources/research-resources/

2
https://www.qualtrics.com/

3
https://www.education.gouv.fr/

4 We do not report on model fit for a one-factor CFA of our measure of transient mood because a CFA with three items and one latent variable is “just-identified.” The fit of such a model is meaningless. Factor loadings ranged from 0.719 to 0.931, with standard errors ranging from 0.009 to 0.015.

5 Here is the original list in French: *amusant*; *stimulant*; *captivant*; *ennuyant*; *frustrant*; *décourageant*; *insolite*.

6 Age and sex were not included in the models because these two variables were essentially unrelated to the outcome of interest.
