# Supplementary material for: Occupational Depression, Cognitive Performance, and Task Appreciation: A Study Based on Raven’s Advanced Progressive Matrices
Source: Front Psychol. 2021 Sep 20;12:695539. doi: 10.3389/fpsyg.2021.695539 (PMC8488105; doi:10.3389/fpsyg.2021.695539)
Supplement: Supplementary file 1 [file Table_1.pdf]

Supplemental Table 1. Summary of hierarchical linear regression analysis—occupational depression predicting cognitive performance.

	Cognitive performance			
	β	*t*	*p*	Adj. R^2
Step 1				.012
Age	-0.049	-1.796	.073	
Sex	0.109	4.022	.000	
Step 2				.018
Age	-0.044	-1.631	.103	
Sex	0.109	4.059	.000	
Pretest mood	-0.085	-3.154	.002	
Step 3				.021
Age	-0.049	-1.817	.069	
Sex	0.104	3.845	.000	
Pretest mood	-0.047	-1.433	.152	
Occupational depression	-0.066	-1.999	.046	

Notes. N = 1,359. Regarding transient mood, higher scores are reflective of a more negative mood. No variance inflation factor exceeded 1.508, suggesting that multicollinearity was not an issue. Sex was coded 0 for women and 1 for men.
